# IGH amplification in patients with B cell lymphoma unclassifiable, with features intermediate between diffuse large B cell lymphoma and Burkitt’s lymphoma

**DOI:** 10.1186/2050-7771-2-9

**Published:** 2014-05-09

**Authors:** Michael Bellone, Ann-Leslie Zaslav, Tahmeena Ahmed, Htien L Lee, Yupo Ma, Youjun Hu

**Affiliations:** 1 Department of Pathology, Stony Brook University Medical Center, Stony Brook, NY 11794, USA; 2 Cytogenetics, Department of Pathology, Stony Brook University Medical Center, Stony Brook, NY 11794, USA

**Keywords:** B-cell lymphoma, unclassifiable, with features intermediate between diffuse large B-cell lymphoma and Burkitt’s lymphoma, IGH, C-MYC, BCL2

## Abstract

B cell lymphoma, unclassifiable, with features intermediate between diffuse large B-cell lymphoma (DLBCL) and Burkitt’s lymphoma (BL) (B-UNC/BL/DLBCL) is a new category of tumors that have features resembling both DLBCL and BL. These tumors have large and medium sized cells with greater irregularity of nuclei and more prominent nucleoli than BL. Approximately 35% to 50% have C-MYC rearrangements, although half are non-immunoglobulin variants. We identified six cases of B-UNC/BL/DLBCL with low-level IGH amplification. Four patients died with a median survival of 7 months (range, 6–20). In conclusion, to our knowledge low-level IGH amplification has not been previously described and should be evaluated for in this patient population.

## Background

The 2008 World Health Organization (WHO) Classification of Tumours of Haematopoietic and Lymphoid Tissues [1] introduced a new diagnosis called B-cell lymphoma, unclassifiable, with features intermediate between diffuse large B-cell lymphoma (DLBCL) and Burkitt’s Lymphoma (BL) (B-UNC/BL/DLBCL). This diagnosis does not describe a distinct entity, but instead represents a heterogeneous category that assists in classifying cases that do not meet the criteria for either classical BL or DLBCL. Tumors placed in this category typically consist of either a mixture of large cells resembling DLBCL and smaller cells resembling BL. Also included in this category are tumors that morphologically resemble either BL or DLBCL, but have atypical immunophenotypic and cytogenetic features [1].

We present six cases with this intermediate phenotype due to their atypical features as demonstrated by analysis with immunohistochemistry, conventional cytogenetic analysis and fluorescence in-situ hybridization (FISH). In addition to already well defined cytogenetic abnormalities seen in the intermediate phenotype, such as C-MYC rearrangements and IGH/C-MYC translocations, these cases each demonstrated low level amplification of the immunoglobulin heavy chain (IGH) gene. To the best of our knowledge, IGH amplification has not yet been reported in patients with B-UNC/BL/DLBCL.

## Materials and methods

### Case selection

A retrospective search covering a 10-year period was performed using our database software (Sunquest CoPathPlus^TM^) with the following key words: ‘Burkitt-like lymphoma’, ‘atypical Burkitt’s’, ‘high-grade lymphoma’, ‘grey-zone lymphoma’, and ‘B-cell lymphoma, unclassifiable’. These cases met the criteria for B-UNC/BL/DLBCL as defined by the 2008 edition of the WHO Classification of Tumors of Haematopoietic and Lymphoid Tissues [1]. Clinical information was obtained by review of corresponding medical records. A second search was performed to determine the baseline incidence of low-level IGH amplification in our patients with lymphoma.

### Tissue sources

Lymph node and/or extranodal tumor biopsies were performed on all patients. Representative formalin-fixed, paraffin-embedded (FFPE) tissue sections were stained with routine hematoxylin and eosin (H&E) for morphologic analysis. Additional samples for select cases were submitted for flow cytometric and cytogenetic analysis, including FISH when possible. The morphology was reviewed by a hematopathologist and a diagnosis of B-UNC/BL/DLBCL was made based on morphologic, immunophenotypic, and cytogenetic analysis with clinical correlation.

### Immunohistochemistry

Immunohistochemical stains were performed on paraffin-embedded sections of the lymph node and/or extranodal tumor biopsies using Bond-maX autostainer (Leica Microsystems, Australia) according to the manufacturer’s protocol. Mouse anti-human CD3, CD5, CD20, CD79a, BCL2, BCL6, Ki-67, and TdT (Ventana Medical Systems®, Oro Valley, AZ) were used as the primary antibodies. Horseradish peroxidase-labeled rabbit anti-mouse polyclonal antibodies were employed to convert the chromogen substrate. All stains were performed with appropriate positive and negative controls.

### Flow cytometric immunophenotyping

Flow cytometric analysis was performed using the FACSCalibur^TM^ 2000 four-color flow cytometer according to the standard protocol (Becton Dickinson, San Jose, CA). Directly conjugated monoclonal antibodies (Becton Dickinson, San Jose, CA) to the following antigens were used for this analysis: CD2, CD3, CD4, CD5, CD7, CD8, CD10, CD19, CD20, CD23, CD38, CD45, CD79a, BCL2, FMC7, HLA-DR, and terminal deoxynucleotidyl transferase (TdT). Polyclonal and monoclonal rabbit anti-human immunoglobulin light chains were obtained (Becton Dickinson, San Jose, CA). All antibodies were conjugated with fluorescein isothiocyanate (FITC), phycoerythrin (PE), allophycocyanin (APC), or PerCP-Cy5.5. The flow cytometric data was analyzed using CellQuest software (Becton-Dickinson, San Jose, CA).

### Cytogenetic studies

Conventional G-banded cytogenetic analysis on 20 metaphase spreads was performed on two cases of tumor tissue and one case of lymph node tissue using methods previously described. The karyotypes were reported according to the 2013 International System for Human Cytogenetic Nomenclature [2]. FISH analysis of 100 to 200 nuclei was performed on fresh tumor or lymph node tissue and/or FFPE tissue sections as previously described [3], using the following probes: LSI IGH(14q32)/BCL2(18q21) Dual Color and LSI IGH(14q32/ MYC(8q24)/CEP 8 Tri-Color Dual Fusion (DF) probes; and LSI MYC(8q24) Dual Color, LSI IGH(14q32) Dual Color, LSI BCL2(18q21) Dual Color, and LSI TCR-alpha/delta(14q11.2) Dual Color Break Apart Rearrangement (BAR) probes. All probes were supplied by Abbot Molecular (Des Plaines, IL). Low-level amplification was defined as three to six copies of a gene locus.

## Results

### Clinical findings

During the 10-year search period, we identified six cases that met the 2008 WHO criteria for B-UNC/BL/DLBCL [1]. During the same period, FISH for IGH was performed on 146 cases of lymphoma and among these, only the six cases of B-UNC/BL/DLBCL showed three or more copies of the IGH locus. The clinical data for these six patients is summarized in Table 1. There were four males and two females with a median age of 54 (range, 4–68). Four patients (cases 2, 3, 5 and 6) presented with extranodal tumors. At presentation, none of the patients had hepatosplenomegaly. Lymphadenopathy was detected in four patients (cases 1, 2, 4 and 6). An elevated LDH level was detected in all patients at presentation, ranging from 265 to 978 U/l, with a median of 750 U/l (normal range: 94–250 U/l). The median leukocyte count at presentation was 8.25 × 10^9^/l, ranging from 3.6 to 11.4 × 10^9^/l (normal range: 4.8 to 10.8 × 10^9^/l). None of the patients presented with central nervous system (CNS) involvement. During the course of disease in two patients (cases 5 and 6), intracerebral lesions were found by computerized tomography (CT) scanning and lymphoma cells were detected in cerebrospinal fluid analysis. Bone marrow and peripheral blood involvement were detected in one patient (case 5) during the course of disease. All patients were treated with intensive chemotherapy regimens (IC) supplemented with rituximab. Three patients (cases 2, 3, and 6) underwent autologous hematopoietic stem cell transplant (HSCT) for treatment of relapses. One patient (case 5) underwent external beam radiation therapy of an extranodal tumor. Four patients (cases 1, 2, 5, and 6) died with a median survival of 7 months (range, 6–20) after progressing to disseminated disease. Two patients (cases 3 and 4) are alive at 105 and 42 months after diagnosis, respectively, at this time, with the former experiencing multiple relapses.

**Table 1:** Summary of clinical, immunophenotypic, and cytogenetic data for all six patients

**Case #**	**Age/Gender**	**Involved sites at presentation**		**Immunophenotype**							**Karyotype**^**1**^	**FISH**^**2**^**(% cells)**	**Therapy**^**3**^	**Outcome/Follow up**
		**Nodal**	**Extranodal**	**BCL2**	**BCL6**	**CD10**	**CD38**	**MUM1**	**sIg**	**Ki-67%**				
1	52/M	Paraaortic, pancreatic, portohepatic, cervical, axillary, mediastinal	NA	+	+	-	NA	NA	λ	100	Complex^4^	IGH x 3 (100%)	IC	DIED/8 Months
2	24/M	Left external iliac	Left femoral neck	+	+	+	NA	+	-	75-80	46,XY	IGH/C-MYC (46%), IGH x 3–4 (51%)	IC, HSCT	DIED/20 Months
3	61/F	NA	Left maxillary sinus	+	+	-	NA	+	κ	100	46,XX	IGH x 3 (20%)	IC, HSCT	ALIVE/105 Months (Multiple Relapses)
4	68/F	Gastrohepatic, periaortic, pericaval, mesenteric	NA	+	+	+	+	NA	κ	95-99	NA	IGH/BCL2 (91.5%), C-MYC x 3–4 (87%), IGH x 3 (42%)	IC	ALIVE/42 Months
5	44/M	NA	Right submandibular space, right lateral neck	+	+	+	+	NA	κ	100	NA	IGH/C-MYC, C-MYC x 3–4, IGH x 3–6, BCL2 x 3–6 (96%)	IC, EBRT	DIED/6 Months
6	4/M	Retroperitoneal, mesenteric	Liver, Omentum	+	+	+	+	NA	κ	95-99	NA	C-MYC x 3 (13%), IGH x 3 (17%)	IC, HSCT	DIED/6 Months

^1^Karyotype: standard cytogenetic evaluation performed using G-banding.

^2^FISH: performed using the following Abbott Molecular (Des Plaines, Ill) probes: LSI IGH(14q32)/BCL2(18q21) Dual Color, LSI IGH(14q32)/MYC(8q24)/CEP8 Tri-Color Dual Fusion, LSI MYC Dual Color Break Apart (8q24), LSI IGH Dual Color Break Apart (14q32), and LSI TCR alpha/delta Dual Color Break Apart (14q11.2) probes.

^3^Therapy: IC, Intensive chemotherapy; HSCT, Hematopoietic Stem Cell Transplant; EBRT, External Beam Radiotherapy.

^4^Complex Karyotype for patient 1: 44,X,-Y,add(3)t(3;22)(p25;q12),t(8;14)(q24;q32),-21.

### Morphologic and immunophenotypic findings

**Figure 1:**
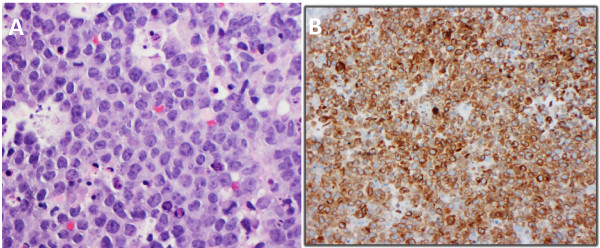
**Morphologic and immunophenotypic findings. (A)** A pelvic mass core biopsy from case 4 showing sheets of medium to large sized abnormal lymphoid cells in a diffuse growth pattern. There is a monotonous appearance to the proliferating cells. A starry-sky pattern with tingible-body macrophages is seen (Hematoxylin-Eosin, original magnification × 400). **(B)** BCL-2 immunohistochemistry from the same case showing diffuse membranous and cytoplasmic staining of tumor cells (original magnification × 200).

Morphologic and immunophenotypic data for all six patients are summarized in Table 1. At low power magnification, each case demonstrated a diffuse growth pattern with complete effacement of normal lymphoid architecture. Each case also showed a high mitotic rate and a “starry sky” pattern. At higher power magnification, each case was composed of a monomorphic population of medium sized cells admixed with finely dispersed nuclear chromatin and inconspicuous nucleoli (Figure 1A). Each case also featured a smaller population of larger centroblastic cells. Necrosis was only seen in case 6. The proliferation index, estimated via Ki-67 immunohistochemical staining, was 75 to 80% in case 2 and greater than 95% in all other cases. Monotypic surface immunoglobulin light chain expression was detected in five cases (κ 4 and λ 1). All cases expressed BCL-2 (Figure 1B) and BCL-6. CD10 expression was detected in four cases.

### Cytogenetic findings

**Figure 2:**
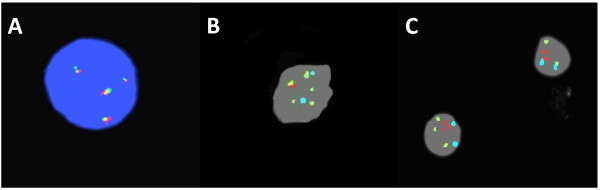
**Cytogenetic findings. (A)** Interphase nuclear FISH from Case 5 using the LSI IGH(14q32) Dual Color Break Apart Rearrangement (BAR) probe (Abbott Molecular Des Plaines, Il) demonstrates amplification of IGH (four fusion signals). **(B)** Interphase nuclear FISH from Case 5 using the LSI IGH(14q32)/MYC(8q24)/Cep8(p11-q11) Tri Color Dual Fusion (DF) probe (Abbott Molecular Des Plaines, Il) demonstrates amplification of IGH (green), a t(8:14)(q24;q32) (red and yellow) and a normal signal pattern for the 8p11-q11 region of chromosome 8 (aqua). **(C)** Interphase nuclear FISH from Case 4 using the same DF probe demonstrates amplification of both IGH (green) and MYC (red) and a normal signal pattern for the 8p11-q11 region of chromosome 8 (aqua).

Cytogenetic data for all six patients are summarized in Table 1. A karyotype was available in only cases 1, 2, and 3. One patient (case 1) had a complex karyotype (44,X,-Y,add(3)t(3;22)(p25;q12),t(8;14)(q24;q32),-21) and the other two were normal. FISH analysis was performed on fresh (cases 1, 2, 3, and 4) and FFPE (cases 1, 2, 3, 5 and 6) tissue. Each patient had three to six copies of IGH (Figure 2A-C), three patients (cases 4, 5, 6) had three to four copies of C-MYC (Figure 2C) and one patient (case 5) had three to six copies of BCL2. One patient (case 4) had an IGH/BCL2 translocation and two patients (cases 2 and 5) had an IGH/C-MYC translocation (Figure 2B). All patients had normal signal patterns for the TCR-alpha/delta and CEP8 loci.

## Discussion

B-UNC/BL/DLBCL represents a heterogeneous group of high grade B-cell lymphomas that cannot be adequately classified as either BL or DLBCL [1]. Cases within this spectrum exhibit features of BL and DLBCL, thus making it difficult to discriminate one from another. In this retrospective study, low-level IGH amplification was identified exclusively in six cases of B-UNC/BL/DLBCL. To the best of our knowledge, this is the first time low-level IGH amplification has been described in association with B-UNC/BL/DLBCL. This novel finding may provide new insight in better classifying these “grey zone” cases.

The morphologic characteristics of B-UNC/BL/DLBCL include features of both BL and DLBCL [1]. Monomorphic medium-sized cells resembling those seen in BL tend to be the predominant cell type in most cases and they are typically associated with a high proliferation rate and a starry-sky pattern. Each case also features a variable amount of larger cells that exhibit nuclear pleomorphism, open vesicular chromatin and multiple nucleoli; all which is more typical of DLBCL. This morphologic heterogeneity was very evident in each case reported in this series. It is one feature that distinguishes intermediate cases from classic BL (Figure 1). The immunophenotype of B-UNC/BL/DLBCL also tends to be variable, expressing germinal center markers such as CD10 and BCL6, but often also expressing BCL2 and typically have a Ki-67 proliferation index (PI) varying between 50% and 95% [1]. Consistent with this, all our cases expressed BCL2 and at least one germinal center marker; five cases expressed at least one post-germinal center marker (i.e. CD38, MUM1) and only one case had a PI less than 95% (Table 1).

To our knowledge, this study is the first to document the presence of IGH amplification in association with B-UNC/BL/DLBCL. Low-level amplification of other genes such as C-MYC, BCL2, and BCL6, has been described. Foot et al. [4] studied 160 cases of non-Burkitt high grade B-cell non-Hodgkin’s lymphoma and found numerous cytogenetic abnormalities by FISH. Three to six copies of C-MYC, BCL2, and BCL6 were detected in 19%, 28%, and 22% of cases, respectively. Kodet et al. [5] re-evaluated 39 cases of previously diagnosed BL or Burkitt-like lymphoma using IHC and FISH. Cytogenetic finding in four cases that were reclassified as B-UNC/BL/DLBCL included a break in the IGH locus and high-level C-MYC amplification. A recent study of 39 cases of B-UNC/BL/DLBCL by Perry et al. [6] found amplification of C-MYC, BCL2, or BCL6 in fourteen cases, either alone or in addition to other cytogenetic findings. Importantly, although each study [4-6] utilized Dual Fusion and/or Break Apart probes specific for IGH, no case with three or more copies was identified.

Three cases in this series also showed low-level C-MYC amplification. B-UNC/BL/DLBCL associated with C-MYC amplification but without IGH/C-MYC translocation or any of its variants is associated with a poor prognosis [7]. Low-level C-MYC amplification in conjunction with BCL2 rearrangements in cases of B-UNC/BL/DLBCL has been previously described [4,6]. Since C-MYC rearrangements occur in 10% of patients with DLBCL, their presence does not change the diagnosis to B-UNC/BL/DLBCL [8]. Similarly, this diagnosis should not be made in cases of otherwise typical BL that lack a detectable C-MYC rearrangement. C-MYC translocations to loci other than an immunoglobulin gene are rarely seen in typical cases of BL, but account for up to half of the C-MYC rearrangements seen in B-UNC/BL/DLBCL [1]. Three cases in our series featured C-MYC rearrangements that were translocated to IGH.

Anywhere from 35 to 50% of B-UNC/BL/DLBCL cases have C-MYC translocations and about 15% of these are accompanied by either BCL2 or BCL6 rearrangements. These “double-hit” lymphomas are among the best characterized subset of B-UNC/BL/DLBCL and carry a poor prognosis [1,9]. Li et al. [10] suggest that either C-MYC rearrangement and BCL2 amplification or BCL2 rearrangement and C-MYC amplification should also be classified as “double hit” since in their series such cases showed poor outcomes similar to cases with both genes rearranged. However, in our series, similar to Perry et al. [6], a patient with C-MYC rearrangement accompanied by BCL2 amplification showed longer survival (case 4, 42 months) than a patient with BCL2 rearrangement and C-MYC amplification (case 5, 6 months). Further study is necessary to determine how these different mechanisms of C-MYC and BCL2 dysregulation influence survival of patients with B-UNC/BL/DLBCL.

Over half of patients with B-UNC/BL/DLBCL present with widespread, often extra-nodal disease. In contrast with BL, these tumors do not show any preferential localization in particular sites, such as the ileocecal region or jaws. Marrow and CNS involvement are not uncommon. B-UNC/BL/DLBCL follows an aggressive course and no appropriate therapeutic approach has been established. The prognosis in our case series was poor since four of six patients in our series died within six to 20 months of initial diagnosis. In a recent study [5], clinical features such as gender, age, and stage were not statistically different between patients who died within four years of diagnosis and those who were alive at four years. In our series, each deceased patient was younger than 55 years of age and progressed to disseminated disease despite receiving multiple cycles of IC regimens. Furthermore, of the three patients who received a HSCT, two died. Studies have shown poor outcomes in patients with B-UNC/BL/DLBCL irrespective of standard DLBCL therapy (i.e. R-CHOP) or a more aggressive approach (i.e. IC regimens or HSCT) [5,11]. One of the surviving patients in our series experienced a relapse five years after diagnosis that was treated with HSCT.

## Conclusions

In conclusion, IGH amplification is a previously undescribed cytogenetic abnormality in patients with B-UNC/BL/DLBCL. The prognosis of B-UNC/BL/DLBCL is known to be poor and this was confirmed in most of our patients. Interestingly, the patient with the longest survival was found to have low-level IGH amplification as its only cytogenetic abnormality. The prognostic significance of low-level amplification of other gene loci such as C-MYC, BCL2, and BCL6 remains uncertain [6]. Therefore, further study is needed to investigate the predictive role of low-level amplification of IGH and other genes in patients with B-UNC/BL/DLBCL.

## Consent

This study did not meet the definition of research by the Institutional Review Board at Stony Brook University Medical Center.

## Competing interests

The authors declare that they have no competing interests.

## Authors’ contributions

MB searched the literature and drafted the manuscript. AZ supplied the cytogenetics data and helped draft the cytogenetics section of manuscript. TA helped draft the manuscript. HL performed the cytogenetic studies. YM supplied the morphologic images and helped draft the manuscript. YH originated the study and helped draft the manuscript. All authors read and approved the final manuscript.
